# Associations of access to exercise opportunities and physical inactivity with cardiovascular–kidney–metabolic mortality: a nationwide ecological study

**DOI:** 10.3389/fpubh.2026.1924014

**Published:** 2026-10-01

**Authors:** Chen Zhao, Zhisheng Zhu, Jiaxian Zhang, Ziyue Ren, Hongwei Liu

**Affiliations:** 1 Department of Plastic Surgery, the First Affiliated Hospital of Jinan University, Guangzhou, China; 2 Institute of New Technology of Plastic Surgery of Jinan University, Guangzhou, China; 3 Key Laboratory of Regenerative Medicine of Ministry of Education, Jinan University, Guangzhou, China

**Keywords:** access to exercise opportunities, cardiovascular-kidney-metabolic syndrome, environmental exposome, physical inactivity, population health

## Abstract

**Objectives:**

To systematically investigate the associations of macro-environmental exposures—specifically, county-level access to exercise opportunities (ATEO)—and population-level physical inactivity (PI) with overall and subtype-specific cardiovascular-kidney-metabolic (CKM) mortality across the United States, and to explore the heterogeneity of these associations across distinct sociodemographic and geographic subgroups.

**Methods:**

We conducted a county-level ecological study using 2014–2019 data from CDC WONDER for CKM mortality and from County Health Rankings for physical activity measures. County-level age-adjusted CKM mortality rates were compared across quartiles of access to exercise opportunities and physical inactivity, with stratification by sex, race, and urbanization. Rate ratios (RRs) for CKM mortality and its three components (cardiovascular, kidney, and metabolic) were estimated using quasi-Poisson generalized linear models, with adjustment for year only (Model 1) or for comprehensive demographic and socioeconomic covariates (Model 2).

**Results:**

A clear geographic gradient emerged: counties in the South had the least exercise access, the highest inactivity levels, and the greatest CKM mortality burden. Greater access to exercise opportunities was inversely associated with total CKM mortality [Q4 vs. Q1, RR,0.89 (0.87–0.90)], but this benefit was not uniform across CKM components—while cardiovascular mortality decreased with better access (RR, 0.89), diabetes and renal failure mortality showed unexpectedly higher rates in high-access counties. Regarding physical inactivity, higher levels were associated with an overall elevation in total CKM mortality [Q4 vs. Q1, RR 1.23 (1.22–1.25)], primarily driven by cardiovascular disease (RR 1.24). However, the associations with the other two components were less consistent: diabetes mortality showed a modest increase (RR 1.23) but with a non-monotonic gradient across quartiles, while renal failure mortality unexpectedly decreased in the highest inactivity quartile. These general patterns were observed across most demographic and geographic subgroups, although the effect magnitudes varied by sex, race, and urbanization.

**Conclusion:**

Our findings demonstrate that limited access to exercise opportunities and higher physical inactivity are associated with elevated total CKM mortality, with cardiovascular disease being the predominant driver. While the associations with diabetes and renal failure showed some heterogeneity, these results support population-wide efforts to reduce physical inactivity and targeted environmental investments in underserved communities as potential strategies to alleviate the combined burden of cardiovascular, diabetic, and renal mortality.

## Introduction

Cardiovascular-kidney-metabolic (CKM) syndrome, a recently formalized construct by the American Heart Association, represents a complex interplay of pathophysiological mechanisms across multiple organ systems and has emerged as a formidable challenge to global public health (1, 2). Driven by population aging and the escalating prevalence of metabolic risk factors, the overlapping burden of cardiovascular disease, chronic kidney disease, and metabolic dysfunction has surged (2). Crucially, CKM syndrome is not merely a superimposition of isolated conditions; rather, it amplifies the trajectory of progressive multi-organ failure and premature mortality through systemic synergistic interactions, thereby challenging traditional, fragmented, single-disease paradigms (3).

For the primary prevention and management of CKM syndrome, lifestyle interventions—particularly regular physical activity—are universally endorsed as highly cost-effective strategies (4–7). Regular physical activity confers robust protection to the cardiovascular and microvascular systems by ameliorating insulin resistance, attenuating systemic inflammation, and preserving endothelial function (1, 8, 9). However, a substantial gap persists between evidence-based clinical recommendations and actual population-level adherence. This deficit in physical activity is not solely a manifestation of inadequate individual health literacy, but is more profoundly constrained by the upstream social determinants of health (SDOH), particularly the macro-built environment (10–13).

The spatial maldistribution of access to exercise opportunities (ATEO)—such as the scarcity of public parks, safe walking trails, and recreational facilities—constitutes a structural barrier that limits residents from adopting active lifestyles. Consequently, this environmental deprivation often translates into high regional rates of physical inactivity (PI) (13, 14). While previous spatial epidemiological studies have established independent associations between the built environment and isolated chronic diseases (e.g., cardiovascular disease or diabetes), few have conceptualized CKM syndrome as an interconnected continuum. Furthermore, the extent to which structural environmental resources relate to the composite and subtype-specific mortality burdens of CKM, and how these effects vary across distinct demographic and geographic subgroups, remain largely unexplored.

To address these critical gaps, we conducted a nationwide ecological analysis to systematically evaluate the associations of county-level ATEO and PI with both overall and subtype-specific CKM mortality across the United States. Given that U.S. counties serve as critical local nodes within the multilevel public health system for resource allocation, county-level analyses are highly informative for structural interventions. We hypothesized that higher ATEO is significantly associated with reduced CKM mortality, while higher PI is associated with elevated mortality risk, and that these associations exhibit heterogeneity across sociodemographic and urban–rural gradients. By comprehensively adjusting for demographic, socio-economic, and broader healthcare contextual factors, while carefully avoiding over-adjustment for metabolic mediators, this study aims to provide population-level empirical evidence to elucidate the role of the macro-built environment in multi-systemic CKM outcomes.

## Methods

### Study design

In this nationwide ecological study, we evaluated the county-level associations between environmental access to exercise opportunities, physical inactivity, and mortality attributable to cardiovascular–kidney–metabolic (CKM) conditions across the United States over the period 2014–2019.

### Data source for mortality outcome

Mortality and population counts for all U.S. counties from 2014 through 2019 were derived from the Detailed Mortality—Underlying Cause of Death database via the CDC WONDER platform.^1^ This database comprehensively compiles death certificates for U.S. residents, identifying a single underlying cause of death alongside decedent demographic characteristics, including sex, race, age, and county of residence. Additionally, county-level urbanization classifications were assigned according to the NCHS Urban–Rural Classification Scheme for Counties to facilitate spatial stratification. For the present study, we restricted the analysis to deaths with an underlying cause coded as cardiovascular disease, diabetes mellitus, or renal failure, collectively defined as CKM mortality (ICD-10 coding details are provided below).

Using the International Classification of Diseases, 10th Revision (ICD-10), we extracted age-adjusted mortality rates and corresponding 95% confidence intervals (CIs) for all deaths attributable to CKM syndrome over the 2014–2019 period. In this study, CKM mortality was defined as deaths with an underlying cause of death coded as cardiovascular disease (CVD), diabetes mellitus, or renal failure, consistent with prior studies and aligned with the AHA CKM stage 4 construct (15, 16). Specifically, overall CVD mortality was defined by the broad ICD-10 range I00-I99 for the composite CKM mortality endpoint. For subsequent subtype-specific analyses, we further extracted deaths coded as ischemic heart disease (I20-I25), heart failure (I50, as well as I11.0 for hypertensive heart disease with heart failure, and I13.0/I13.2 for hypertensive heart and kidney disease with heart failure), cerebrovascular diseases (I60-I69, including acute stroke I60-I64 and sequelae I69), atrial fibrillation (I48), and peripheral artery disease (I70.2, I73.9). Diabetes mellitus was defined by codes E10-E14. Renal failure was defined by codes N17-N19, with specific inclusion of end-stage renal disease (N18.6). All mortality rates are expressed per 100,000 adults.

For subgroup analyses, deaths and population counts were stratified by sex (male vs. female), race (White, American Indian or Alaska Native, and Black or African American), and urbanization level (Large Fringe Metro, Medium Metro, Micropolitan [Nonmetro], NonCore [Nonmetro], and Small Metro).

### Exposures

County-level estimates of physical activity–related exposures—specifically, access to exercise opportunities and physical inactivity—were obtained from the County Health Rankings & Roadmaps (CHR&R) program.^2^ CHR&R is an annual compilation of county-level health data derived from various national and state sources, providing validated measures of health outcomes and health factors for nearly all U.S. counties. For the present study, we extracted county-level data on access to exercise opportunities and physical inactivity for the period corresponding to our mortality data (2014–2019). These measures have been widely used and validated in spatial epidemiological research (15, 17, 18).

### Access to exercise opportunities

Access to exercise opportunities was defined as the county-level percentage of residents living within a reasonable distance of parks or recreational facilities. Based strictly on the CHR&R methodology, a “reasonable distance” was operationalized as residing within half a mile of a park, within one mile of a recreational facility in urban areas, or within three miles of such a facility in rural areas. As detailed by the CHR&R methodology, this metric was generated by integrating park boundaries from the Esri USA Parks dataset, recreational facilities identified by Standard Industrial Classification codes (derived from the Esri Business Summary and YMCA locations), and 2020 census block population counts using ArcGIS Pro. Adequate access was initially evaluated at the census block level based on these precise proximity thresholds; the populations of the qualifying blocks were then aggregated to calculate the final county-level percentage (ranging from 0 to 1, where a higher score denoted more favorable access). We then converted these index values into percentile ranks and divided them into quartiles according to the national distribution: quartile 1 (0–0.25, representing the least access) through quartile 4 (0.75–1.00, representing the most access). It is worth noting that this measure does not encompass all possible exercise venues (e.g., sidewalks, shopping malls, school gyms), and proximity alone does not fully reflect barriers such as user fees or variations in park quality and amenities.

### Physical inactivity

Physical inactivity was derived from self-reported data from the Behavioral Risk Factor Surveillance System (BRFSS), a state-based telephone survey of non-institutionalized US adults aged 18 years and older. It was defined as the age-adjusted percentage of respondents who reported engaging in no leisure-time physical activity during the past month, excluding routine job-related activities. The county-level estimates were generated by the CDC using multilevel statistical modeling (PLACES project), which incorporated individual-level demographic factors and county-level contextual characteristics to produce reliable small-area estimates even for counties with limited survey samples. The resulting index for each county ranged from 0 to 0.5, with higher scores reflecting greater levels of physical inactivity. We subsequently converted these index values into percentile ranks and categorized them into quartiles according to the national distribution: quartile 1 (0–0.125, denoting the lowest inactivity) through quartile 4 (0.375–0.500, denoting the highest inactivity). It should be noted that this measure does not account for physical activity undertaken as part of paid or unpaid labor. Given that many physically demanding jobs are concentrated in lower-income communities, this omission may lead to certain populations being inaccurately labeled as physically inactive.

### Covariates

To minimize potential confounding, we adjusted for a set of county-level covariates sourced from the County Health Rankings and Roadmaps dataset. These covariates were selected based on their established associations with CKM conditions and were incorporated into both subgroup classifications and multivariable regression models. They comprised two broad categories: (1) demographic characteristics, including the percentages of female residents, Hispanic residents, rural population, and population aged 65 years or older; and (2) socioeconomic and health-related factors, including the percentage of uninsured residents, the population-to-primary-care-physician ratio, household income inequality, smoking prevalence, preventable hospital stay rate, and food environment index.

### Statistical analysis

We restricted our main analyses to counties with complete records for access to exercise opportunities, physical inactivity, and CKM-related mortality. Counties with missing data for any of these core variables were removed to preserve the integrity of the analytical results. No missingness was detected in the covariate dataset. Ultimately, 3,028 of the 3,235 U.S. counties (approximately 93.6%) met the inclusion criteria and were retained for the final analysis.

County-level geographic boundary files were obtained from the 2024 cartographic boundary shapefile released by the U.S. Census Bureau and were directly imported into the R environment via the tigris package for spatial data handling and map generation. To evaluate the crude associations, we employed generalized linear models (GLMs) to compare CKM mortality rates across quartile categories of the two physical activity metrics. We further performed subgroup analyses stratified by demographic attributes (sex and race) and urbanization levels to examine group-specific mortality patterns.

We calculated rate ratios (RRs) with corresponding 95% confidence intervals (CIs) by contrasting CKM mortality rates—both for the composite outcome and its individual components—between the highest (fourth) and lowest (first) quartiles of the exposure distributions. A quasi-Poisson GLM specification was adopted, incorporating the logarithm of county population as an offset term to account for overdispersion and to appropriately model event rates relative to population size. Two sequential models were constructed: Model 1 was adjusted for calendar year only, while Model 2 represented a fully adjusted specification that additionally included a comprehensive set of county-level covariates, namely the percentages of females, Hispanic residents, rural population, and adults aged 65 years or older, along with the uninsured rate, population-to-primary-care-physician ratio, household income inequality, smoking prevalence, preventable hospital stay rate, and food environment index.

To formally evaluate the potential multicollinearity between the two core exposure variables (ATEO and PI), we calculated Pearson and Spearman correlation coefficients and derived the variance inflation factor (VIF). A VIF value below 5 was considered indicative of no substantial collinearity, justifying the simultaneous inclusion of both exposures in the regression models.

To further evaluate the consistency of our findings across diverse population subgroups, we conducted a series of stratified analyses. Counties were categorized by their predominant demographic and geographic characteristics, including sex composition, urbanization level (Large Fringe Metro, Medium Metro, Micropolitan (Nonmetro), NonCore (Nonmetro), and Small Metro), and racial composition (White, American Indian or Alaska Native, and Black or African American). These subgroup analyses were performed separately for each exposure metric (access to exercise opportunities and physical inactivity) to examine whether the observed associations with CKM mortality varied across strata.

To further characterize the shape of the dose–response relationships without imposing a predefined categorical structure, we performed a complementary sensitivity analysis using restricted cubic splines (RCS) with three knots (located at the 10th, 50th, and 90th percentiles) within negative binomial regression models. In these RCS models, the exposure variables (ATEO and PI) were treated as continuous percentile ranks. The models were adjusted for the same set of county-level covariates as Model 2 (i.e., percentage of female residents, percentage of Hispanic residents, percentage of rural population, percentage of adults aged 65 years or older, uninsured rate, population-to-primary-care-physician ratio, income inequality, smoking prevalence, preventable hospital stay rate, and food environment index). County-level clustering was accounted for using robust standard errors. Non-linearity was formally tested by examining the null hypothesis that the coefficients of all spline terms were jointly equal to zero. The RCS results were visualized as dose–response curves, with the median exposure value serving as the reference.

To address potential overdispersion and spatial dependence inherent in county-level mortality data, we adopted negative binomial regression models (glm.nb in R) with county-level clustered robust standard errors (via vcovCL from the sandwich package). The dispersion parameter (theta) was estimated for each model to confirm appropriate handling of extra-Poisson variation. We further assessed residual spatial autocorrelation using Moran’s I test on the Pearson residuals. As a sensitivity analysis, we refit the models using a spatial error specification (SEM) to evaluate the robustness of our effect estimates to spatial dependence.

## Results

### Access to exercise opportunities and CKM mortality

Figure 1 illustrates the county-level distributions of access to exercise opportunities (ATEO), physical inactivity (PI), and age-adjusted CKM mortality rates (AAMR) in the USA for 2014 and 2019. A clear geographic gradient emerged. Quantitatively, the South had the highest proportion of counties with limited access to exercise opportunities (ATEO Q1: 45.1% in 2014 and 25.1% in 2019), the highest proportion of counties with elevated physical inactivity (PI Q4: 43.6% in 2014 and 28.6% in 2019), and the highest proportion of counties with elevated CKM mortality (AAMR Q4: 39.9% in 2014 and 35.6% in 2019), compared with all other U.S. Census regions (Supplementary Table S2). Conversely, the northeastern and western regions generally showed more favorable profiles across all three metrics.

**Figure 1:**
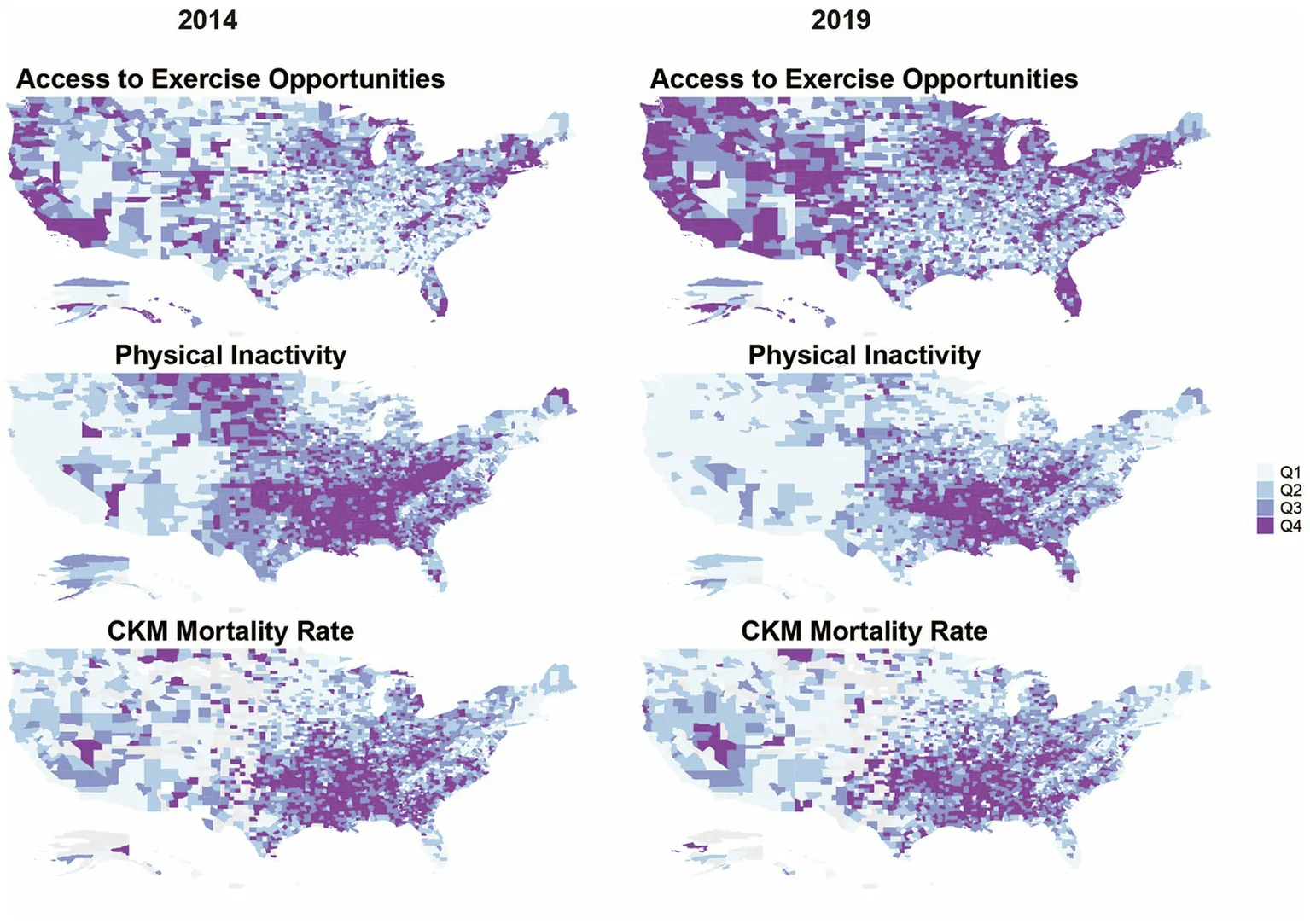
Spatiotemporal distribution of county-level physical activity metrics and cardiovascular-kidney-metabolic (CKM) mortality across the United States, comparing 2014 and 2019.

As presented in Table 1, the age-adjusted total CKM mortality rates (per 100,000 population) were highest in the first access quartile [887.95 (95% CI, 884.60–891.30)] and lowest in the fourth quartile [662.52 (95% CI, 661.84–663.20)]. A consistent stepwise decline in CKM mortality from the lowest to the highest quartile of exercise opportunities was observed across all sociodemographic subgroups, including age, sex, race, and urbanization (all P for trend < 0.001). Notably, the magnitude of risk reduction conferred by higher ATEO was more pronounced in the 45–64 age group compared to those aged ≥65 years. When stratified by urbanization level, the rate ratios (RRs) for CKM mortality comparing the fourth versus first quartile showed modest heterogeneity; notably, residents in large fringe metro areas experienced a 33% lower risk [RR, 0.67 (0.51–0.89)], whereas those in noncore areas exhibited a more modest 9% reduction (RR, 0.91 [0.85–0.98]) (Table 1). After full adjustment for sociodemographic covariates, the county-level access to exercise opportunities remained significantly protective against CKM mortality [Q4 vs. Q1, RR, 0.89 (0.87–0.90)] (Figure 2). In terms of absolute risk, the age-adjusted mortality rate difference (ARD) comparing counties in the highest versus lowest quartile of ATEO was −78.81 deaths per 100,000 population (Supplementary Table S3). Overall, the protective association was observed in most subgroup analyses stratified by sex, urbanization, and race, although estimates varied across some strata (Supplementary Figures S1–S3). These results generally support the main finding, while acknowledging certain subgroup heterogeneity.

**Table 1:** Age-adjusted mortality rates for cardiovascular-kidney-metabolic syndrome, by quartile of county-level access to exercise opportunities, U.S. counties, 2014–2019.

Cardio-kidney-metabolic mortality	Access to exercise opportunities						
	Q1	Q2	Q3	Q4	*p* value for trend*	Rate ratio quartile 4/1***	*p* value*
Total	887.95 (884.6, 891.3)	819.5 (817.34, 821.66)	761.54 (759.99, 763.09)	662.52 (661.84, 663.2)	<0.0001	0.89 (0.87, 0.90)	<0.0001
Age							
45–64y	292.18 (289.17, 295.2)	252.05 (250.33, 253.78)	217.51 (216.39, 218.63)	165.85 (165.43, 166.26)	<0.0001	0.5 (0.48, 0.52)	<0.0001
>64	1934.8 (1927.24, 1942.38)	1816.59 (1811.47, 1821.72)	1717.49 (1713.71, 1721.28)	1535.25 (1533.53, 1536.97)	<0.0001	0.81 (0.77, 0.85)	<0.0001
Sex							
Female	713.36 (708.85, 717.88)	683.22 (680.33, 686.12)	640.66 (638.64, 642.68)	550 (549.18, 550.81)	<0.0001	0.49 (0.45, 0.53)	<0.0001
Male	1072.46 (1066.03, 1078.91)	995.92 (992.03, 999.82)	923.69 (920.99, 926.4)	805.85 (804.68, 807.02)	<0.0001	0.58 (0.55, 0.62)	<0.0001
Race							
American Indian or Alaska Native	477.15 (458.61, 496.05)	342.96 (327.13, 359.16)	98.57 (90.05, 107.48)	237.57 (230.22, 245.03)	<0.0001	0.37 (0.35, 0.39)	<0.0001
Black or African American	1066.12 (1051.3, 1081.04)	1030.01 (1019.05, 1041.04)	1003.05 (995.28, 1010.84)	901.29 (898.76, 903.83)	<0.0001	0.67 (0.63, 0.71)	<0.0001
White	858.42 (854.77, 862.08)	801.25 (798.98, 803.52)	745.47 (743.86, 747.09)	648.39 (647.65, 649.13)	<0.0001	0.59 (0.55, 0.62)	<0.0001
Urbanization							
Large Fringe Metro	826.54 (816.05, 837.1)	800.66 (794.25, 807.1)	715.02 (711.24, 718.81)	635.09 (633.87, 636.3)	<0.0001	0.67 (0.51, 0.89)	0.005
Medium Metro	820.82 (812.03, 829.65)	764.3 (759.74, 768.89)	745.17 (742.26, 748.07)	669.17 (667.7, 670.65)	<0.0001	0.74 (0.61, 0.91)	0.004
Micropolitan (Nonmetro)	906.37 (898.87, 913.9)	860.09 (855.93, 864.25)	806.77 (803.39, 810.16)	711.56 (707.13, 716.01)	<0.0001	0.85 (0.77, 0.93)	0.001
NonCore (Nonmetro)	921.41 (916.12, 926.72)	869.31 (864.09, 874.54)	781.64 (775.35, 787.94)	730 (721.47, 738.58)	0.008	0.91 (0.85, 0.98)	0.009
Small Metro	852.11 (842.93, 861.33)	811.07 (805.55, 816.6)	763.46 (760.26, 766.66)	668.26 (665.48, 671.06)	<0.0001	0.73 (0.63, 0.85)	<0.0001

Values are age-adjusted mortality rates per 100,000 adults, with 95% confidence intervals (CIs) provided in parentheses.

*p value for trend was derived from generalized linear models (GLMs) by treating the quartile categories as a continuous ordinal variable, testing for a linear gradient in CKM mortality rates across increasing levels of exercise access (from Q1 to Q4).

**p value presented alongside the rate ratio was obtained from the same GLMs, specifically testing the null hypothesis that the rate ratio equals 1.00 (i.e., no difference in mortality risk between the highest and lowest quartiles of exercise access).

***All models were adjusted for the following covariates: year, percentage of female, percentage of Hispanic, percentage of rural population, percentage of population over the age of 65 years, percentage of uninsured population, ratio of population to primary care physician, household income inequality, adults smoking prevalence, preventable hospital stay rate, and food environment index. This adjustment set corresponds to the fully adjusted specification (Model 2) employed in the subsequent regression analyses.

**Figure 2:**
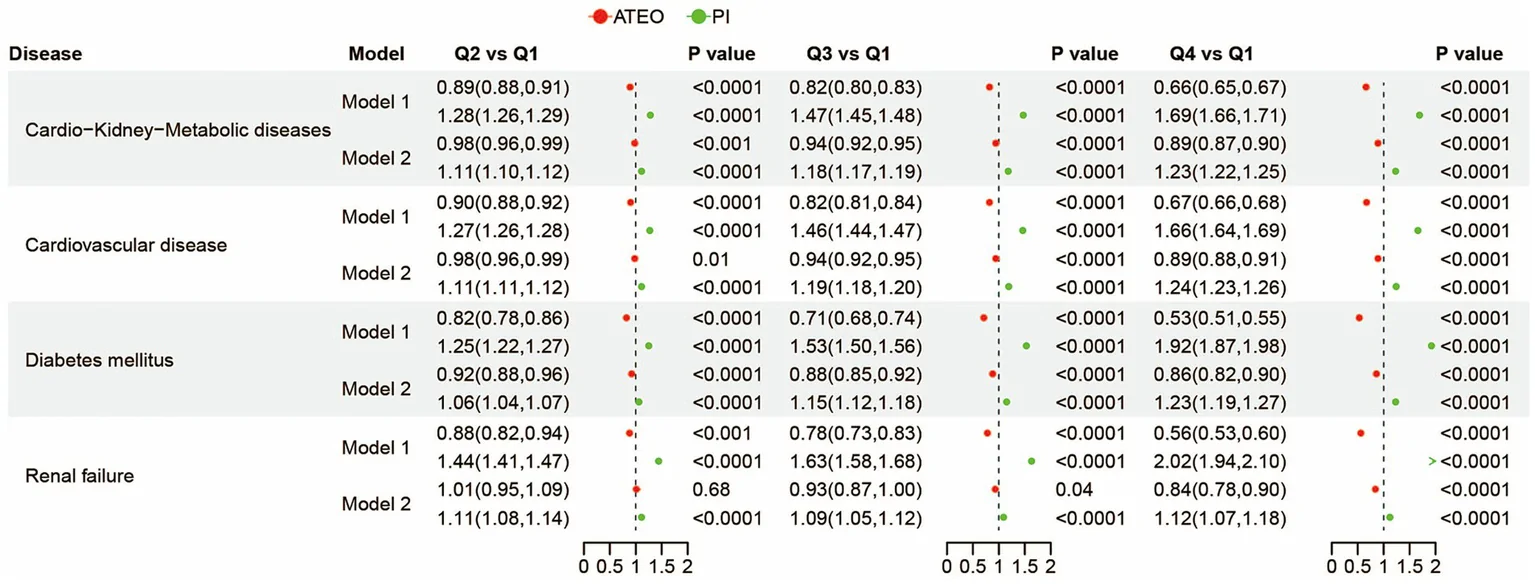
Rate ratios and 95% confidence intervals for the association of access to exercise opportunities and physical inactivity with age-adjusted mortality from specific CKM syndrome components. Estimates compare counties in the highest (4th) quartile to those in the lowest (1st) quartile as the reference. Model 1 was adjusted for year. Model 2 was additionally adjusted for percentage of female, percentage of Hispanic, percentage of rural population, percentage of population over the age of 65 years, percentage of uninsured population, ratio of population to primary care physician, household income inequality, adults smoking prevalence, preventable hospital stay rate, and food environment index.

When dissecting the composite CKM mortality into its specific constituents, heterogeneous patterns across quartiles of access to exercise opportunities were observed. For cardiovascular disease, the age-adjusted mortality rate exhibited a decreasing gradient, from 763.30 (760.11–766.50) per 100,000 in the lowest quartile (Q1) to 576.06 (575.42–576.69) in the highest quartile (Q4). In contrast, renal failure mortality showed a striking upward trend, rising from 6.17 (5.71–6.65) in Q1 to 34.10 (33.92–34.28) in Q4, while diabetes mellitus mortality initially increased from 23.75 (22.86–24.65) in Q1 to a peak of 63.05 (62.38–63.72) in Q3, followed by a modest reduction to 55.01 (54.79–55.22) in Q4 (Table 2). After comprehensive adjustment for demographic and socioeconomic covariates (Model 2), greater access to exercise opportunities was significantly protective across all outcomes, yielding RRs of 0.89 (0.87–0.90) for total CKM, 0.89 (0.88–0.91) for cardiovascular disease, 0.84 (0.78–0.90) for renal failure, and 0.86 (0.82–0.90) for diabetes mellitus, respectively (Figure 2). Moreover, when further stratifying the cardiovascular disease component by its clinical subtypes (e.g., ischemic heart disease, acute myocardial infarction, heart failure, and cerebrovascular diseases), the direction and magnitude of the associations remained broadly consistent, with the exception of atrial fibrillation, demonstrating the robustness of the protective effect of exercise access across most CVD subtypes (Supplementary Table S1; Supplementary Figure S4).

**Table 2:** Age-adjusted mortality rates for specific CKM syndrome components, by quartile of access to exercise opportunities.

Cardio-kidney-metabolic mortality	Access to exercise opportunities						
	Q1	Q2	Q3	Q4	*p* value for trend*	Rate ratio quartile 4/1***	*p* value**
Cardio-kidney-metabolic diseases	887.95 (884.6, 891.3)	819.5 (817.34, 821.66)	761.54 (759.99, 763.09)	662.52 (661.84, 663.2)	<0.0001	0.89 (0.87, 0.90)	<0.0001
Cardiovascular disease	763.3 (760.11, 766.5)	707.3 (705.27, 709.34)	658.36 (656.91, 659.81)	576.06 (575.42, 576.69)	<0.0001	0.89 (0.88, 0.91)	<0.0001
Diabetes mellitus	23.75 (22.86, 24.65)	51.99 (51.05, 52.93)	63.05 (62.38, 63.72)	55.01 (54.79, 55.22)	<0.0001	0.86 (0.82, 0.90)	<0.0001
Renal failure	6.17 (5.71, 6.65)	20.81 (20.18, 21.44)	34.07 (33.54, 34.6)	34.1 (33.92, 34.28)	<0.0001	0.84 (0.78, 0.90)	0.002

Values are age-adjusted mortality rates per 100,000 adults, with 95% confidence intervals (CIs) provided in parentheses.

*p value for trend was derived from generalized linear models (GLMs) by treating the quartile categories as a continuous ordinal variable, testing for a linear gradient in CKM mortality rates across increasing levels of exercise access (from Q1 to Q4).

**p value presented alongside the rate ratio was obtained from the same GLMs, specifically testing the null hypothesis that the rate ratio equals 1.00 (i.e., no difference in mortality risk between the highest and lowest quartiles of exercise access).

***All models were adjusted for the following covariates: year, percentage of female, percentage of Hispanic, percentage of rural population, percentage of population over the age of 65 years, percentage of uninsured population, ratio of population to primary care physician, household income inequality, adults smoking prevalence, preventable hospital stay rate, and food environment index. This adjustment set corresponds to the fully adjusted specification (Model 2) employed in the subsequent regression analyses.

### Physical inactivity and CKM mortality

As shown in Table 3, the age-adjusted total CKM mortality rate (per 100,000 population) was lowest in the first physical inactivity quartile [624.76 (624.01–625.51)] and highest in the fourth quartile [926.79 (924.30–929.28)]. County-level age-adjusted CKM mortality rates were compared across quartiles of access to exercise opportunities and physical inactivity, with stratification by age, sex, race, and urbanization. The rate ratios (RRs) comparing the fourth versus first quartile demonstrated modest heterogeneity when stratified by sex, race, and urbanization. Specifically, males in the highest inactivity quartile experienced a 1.75-fold higher CKM mortality [RR, 1.75 (1.67–1.84)] compared with those in the lowest quartile, whereas the corresponding RR for females was 1.93 (1.82–2.05). White adults in the fourth quartile had a significantly elevated risk of CKM mortality relative to the first quartile [RR, 1.68 (1.60–1.76)]. Geographically, residents in small metro areas exhibited a 1.40-fold higher risk [RR, 1.40 (1.26–1.56)] when comparing the fourth versus first inactivity quartile (Table 3). In the crude model (Model 1), the RR for CKM mortality comparing the fourth to the first quartile of physical inactivity was 1.69 (1.66–1.71). After full covariate adjustment (Model 2), the RR attenuated to 1.23 (1.22–1.25) (Figure 2). The corresponding absolute rate difference (ARD) was +112.38 deaths per 100,000 population, indicating that counties with the highest inactivity levels experienced over 100 additional CKM deaths per 100,000 people compared with the least inactive counties (Supplementary Table S3). Subgroup analyses stratified by sex, urbanization, and race broadly supported the primary findings, though the magnitude and significance of the associations differed somewhat across subgroups (Supplementary Figures S1–S3).

**Table 3:** Age-adjusted mortality rates for CKM syndrome, by quartile of county-level physical inactivity prevalence, U.S. counties, 2014–2019.

Cardio-kidney-metabolic mortality	Physical inactivity						
	Q1	Q2	Q3	Q4	*p* value for trend*	Rate ratio quartile 4/1***	*p* value**
Total	624.76 (624.01, 625.51)	739.96 (738.75, 741.18)	817.91 (816.15, 819.67)	926.79 (924.3, 929.28)	<0.0001	1.23 (1.22, 1.25)	<0.0001
Age							
45–64y	146.85 (146.4, 147.3)	203.5 (202.68, 204.33)	245.12 (243.83, 246.42)	318.14 (316.07, 320.21)	<0.0001	2.29 (2.24, 2.35)	<0.0001
>64	1464.51 (1462.6, 1466.43)	1682.6 (1679.58, 1685.62)	1824.39 (1820.1, 1828.68)	1996.28 (1990.46, 2002.11)	<0.0001	1.32 (1.28, 1.37)	<0.0001
Sex							
Female	518.17 (517.27, 519.08)	614.08 (612.59, 615.56)	681.03 (678.81, 683.25)	775.13 (771.71, 778.56)	<0.0001	1.93 (1.82, 2.05)	<0.0001
Male	759.15 (757.85, 760.44)	902.32 (900.21, 904.44)	993.74 (990.62, 996.86)	1133.71 (1129.08, 1138.35)	<0.0001	1.75 (1.67, 1.84)	<0.0001
Race							
American Indian or Alaska Native	271.57 (263.27, 279.99)	285.68 (274.25, 297.35)	200.68 (188.04, 213.71)	322.35 (305.29, 339.86)	<0.0001	1.39 (1.3, 1.48)	<0.0001
Black or African American	863.01 (859.63, 866.39)	934.79 (930.59, 939)	988.86 (983.02, 994.71)	1087.46 (1078.23, 1096.72)	<0.0001	1.48 (1.43, 1.54)	<0.0001
White	618.44 (617.63, 619.25)	718.49 (717.19, 719.79)	797.8 (795.88, 799.71)	904.57 (901.84, 907.3)	<0.0001	1.68 (1.6, 1.76)	<0.0001
Urbanization							
Large Fringe Metro	606.13 (604.76, 607.49)	704.88 (702.51, 707.26)	781.93 (777.69, 786.19)	868.02 (860.19, 875.9)	<0.0001	1.52 (1.24, 1.87)	<0.0001
Medium Metro	619.37 (617.7, 621.04)	734.16 (731.82, 736.51)	817.5 (813.92, 821.08)	878.23 (872.06, 884.42)	<0.0001	1.44 (1.25, 1.66)	<0.0001
Micropolitan (Nonmetro)	665.49 (661.76, 669.24)	778.43 (774.04, 782.83)	856.53 (851.97, 861.11)	949.24 (944.63, 953.86)	<0.0001	1.24 (1.16, 1.33)	<0.0001
NonCore (Nonmetro)	658.77 (652.72, 664.85)	755.83 (750.1, 761.58)	848.64 (842.77, 854.54)	989.81 (984.37, 995.27)	<0.0001	1.22 (1.15, 1.29)	<0.0001
Small Metro	625.33 (622.61, 628.07)	736.55 (732.75, 740.37)	846.32 (841.7, 850.95)	914.85 (908.64, 921.08)	<0.0001	1.4 (1.26, 1.56)	<0.0001

Values are age-adjusted mortality rates per 100,000 adults, with 95% confidence intervals (CIs) provided in parentheses.

*p value for trend was derived from generalized linear models (GLMs) by treating the quartile categories as a continuous ordinal variable, testing for a linear gradient in CKM mortality rates across increasing levels of physical inactivity (from Q1 to Q4).

**p value presented alongside the rate ratio was obtained from the same GLMs, specifically testing the null hypothesis that the rate ratio equals 1.00 (i.e., no difference in mortality risk between the highest and lowest quartiles of physical inactivity).

***All models were adjusted for the following covariates: year, percentage of female, percentage of Hispanic, percentage of rural population, percentage of population over the age of 65 years, percentage of uninsured population, ratio of population to primary care physician, household income inequality, adults smoking prevalence, preventable hospital stay rate, and food environment index. This adjustment set corresponds to the fully adjusted specification (Model 2) employed in the subsequent regression analyses.

When examining the individual components of the composite CKM mortality across quartiles of physical inactivity, divergent patterns emerged. For cardiovascular disease, the age-adjusted mortality rate increased progressively from 543.49 (542.79–544.20) per 100,000 in the lowest physical inactivity quartile (Q1) to 800.33 (797.98–802.69) in the highest quartile (Q4). Similarly, diabetes mellitus mortality showed an upward trajectory, rising from 52.67 (52.43–52.92) in Q1 to a peak of 61.80 (61.15–62.45) in Q3, with a slight attenuation to 55.36 (54.31–56.43) in Q4. In stark contrast, renal failure mortality exhibited a declining gradient, decreasing from 30.56 (30.37–30.76) in Q1 to 20.78 (20.09–21.48) in Q4 (Table 4). After full adjustment for demographic and socioeconomic covariates (Model 2), higher physical inactivity was significantly associated, comparing the fourth to the first quartile, with elevated CKM and cardiovascular mortality, yielding RRs of 1.23 (1.22–1.25) and 1.24 (1.23–1.26), respectively; for renal failure and diabetes mellitus, the corresponding RRs were 1.12 (1.07–1.18) and 1.23 (1.19–1.27), respectively (Figure 2). Aside from atrial fibrillation, the adverse associations of physical inactivity were observed across most examined cardiovascular subtypes, further supporting the overall findings (Supplementary Table S1; Supplementary Figure S4).

**Table 4:** Age-adjusted mortality rates for specific CKM syndrome components, by quartile of physical inactivity.

Cardio-kidney-metabolic mortality	Physical inactivity						
	Q1	Q2	Q3	Q4	*p* value for trend*	Rate ratio quartile 4/1***	*p* value**
Cardio-kidney-metabolic diseases	624.76 (624.01, 625.51)	739.96 (738.75, 741.18)	817.91 (816.15, 819.67)	926.79 (924.3, 929.28)	<0.0001	1.23 (1.22, 1.25)	<0.0001
Cardiovascular disease	543.49 (542.79, 544.2)	640.51 (639.38, 641.65)	707.75 (706.1, 709.4)	800.33 (797.98, 802.69)	<0.0001	1.24 (1.23, 1.26)	<0.0001
Diabetes mellitus	52.67 (52.43, 52.92)	57.78 (57.37, 58.19)	61.8 (61.15, 62.45)	55.36 (54.31, 56.43)	<0.0001	1.23 (1.19, 1.27)	<0.0001
Renal failure	30.56 (30.37, 30.76)	38.65 (38.31, 38.99)	33.14 (32.64, 33.64)	20.78 (20.09, 21.48)	<0.0001	1.12 (1.07, 1.18)	<0.0001

Values are age-adjusted mortality rates per 100,000 adults, with 95% confidence intervals (CIs) provided in parentheses.

*p value for trend was derived from generalized linear models (GLMs) by treating the quartile categories as a continuous ordinal variable, testing for a linear gradient in CKM mortality rates across increasing levels of physical inactivity (from Q1 to Q4).

**p value presented alongside the rate ratio was obtained from the same GLMs, specifically testing the null hypothesis that the rate ratio equals 1.00 (i.e., no difference in mortality risk between the highest and lowest quartiles of physical inactivity).

***All models were adjusted for the following covariates: year, percentage of female, percentage of Hispanic, percentage of rural population, percentage of population over the age of 65 years, percentage of uninsured population, ratio of population to primary care physician, household income inequality, adults smoking prevalence, preventable hospital stay rate, and food environment index. This adjustment set corresponds to the fully adjusted specification (Model 2) employed in the subsequent regression analyses.

### Sensitivity analyses and model diagnostics

Before conducting the sensitivity analyses, we first examined the collinearity between ATEO and PI. The Pearson correlation coefficient was −0.498, and the Spearman rank correlation was −0.524 (both *p* < 0.001), indicating a moderate inverse association. The corresponding VIF was 1.33, well below the conventional threshold of 5, confirming that multicollinearity did not materially affect our estimates.

To evaluate the robustness of our modeling strategy and to formally address potential overdispersion and spatial dependence, we conducted a series of diagnostic tests and sensitivity analyses using negative binomial models with county-level clustered robust standard errors.

The dispersion parameters (theta) were 42.07 for the physical inactivity model and 35.93 for the access-to-exercise model, with Pearson dispersion statistics close to 1.0, confirming that the negative binomial specification appropriately accounted for extra-Poisson variation (Supplementary Table S3).

The Moran’s I test on model residuals yielded a value of 0.219 (*p* < 0.001), indicating significant residual spatial clustering across counties. To assess whether this spatial dependence could materially bias our effect estimates, we refit the models using a spatial error specification (SEM). In the SEM sensitivity analysis (Supplementary Table S3), the coefficients remained consistent with the primary models: for physical inactivity, the coefficient was 0.116 (*p* < 0.001); for access to exercise, the coefficient was −0.018 (*p* = 0.077). Although the precision for the exercise access estimate was slightly attenuated under this more conservative specification (spatial error model), the direction and magnitude of the effect remained consistent with the primary quasi-Poisson models (Q4 vs. Q1 RR = 0.89, 95% CI: 0.87–0.90). In the RCS sensitivity analyses, the dose–response curve for ATEO demonstrated a largely monotonic inverse association with CKM mortality across most of its distribution, with no significant departure from linearity (P for non-linearity > 0.05; Supplementary Figure S5). For PI, the curve revealed a steeper increase in CKM mortality risk at lower to moderate levels of inactivity, which appeared to plateau at higher levels, although the formal test for non-linearity was not statistically significant (*p* > 0.05; Supplementary Figure S6). These supplementary analyses corroborated our primary quartile-based findings, confirming that the observed associations were not artifacts of the chosen categorization scheme. These diagnostic checks collectively support the robustness of our primary findings to spatial confounding and model misspecification. A comprehensive summary of these diagnostic statistics is provided in Supplementary Table S3.

## Discussion

Currently, the public health burden of CKM syndrome is escalating; nearly 90% of US adults are at CKM stage 1 or higher, and over 15% have progressed to advanced stages characterized by a high incidence of cardiovascular events (1, 2, 19). The pathological intertwining of the cardiovascular, renal, and metabolic systems is not merely a simple superposition of individual diseases (20–22); rather, synergistic damage across target organs significantly amplifies the risk of progressive multi-organ failure and premature death, posing systemic challenges to traditional single-disease prevention and treatment models (1, 23). Although our findings indicate that cardiovascular disease is the predominant driver of CKM-related mortality, maintaining the integrated CKM framework is crucial, as metabolic and renal dysfunctions synergistically amplify cardiovascular risk, rendering isolated analyses insufficient. Previous research has well established positive associations between urban environmental attributes (e.g., net residential density, public transit, and park distribution) and moderate-to-vigorous physical activity (MVPA) (10, 11, 17). Building upon this, our nationwide county-level ecological analysis demonstrates that high ATEO is significantly and independently associated with lower mortality from overall CKM syndrome and its individual disease subtypes (cardiovascular disease, diabetes, and renal failure), whereas PI is significantly correlated with elevated mortality risks. These robust associations were further corroborated by evaluating the continuous, non-linear dose–response relationships using restricted cubic splines (24). These findings highlight that a supportive macro-built environment is significantly associated with a lower multi-systemic mortality burden of CKM syndrome at the population level.

Notably, as reflected by the crude mortality rates (Tables 2, 4), in the unadjusted models, areas with high ATEO initially exhibited a positive association with microvascular disease (both renal failure and diabetes) mortality. Concurrently, PI displayed an inverse association specifically with renal failure mortality. This initial divergence highlights the complex socio-spatial dynamics of urbanization. Regions with high exercise accessibility—typically highly urbanized areas with well-developed physical infrastructure—are also often accompanied by an “urban health penalty.” This includes severe social stratification, high psychosocial stress, and the spatial clustering of vulnerable populations with higher baseline metabolic risks (25, 26). Specifically, chronic exposure to urban psychosocial stress triggers neuroendocrine dysregulation and elevates allostatic load, which directly accelerates microvascular endothelial dysfunction (27, 28). Furthermore, in such high-stress environments, physical inactivity (PI) exacerbates psychological burdens by depriving vulnerable populations of the stress-buffering and neuroendocrine-regulating benefits of exercise (29). This interplay creates a vicious cycle that synergistically amplifies the mortality risk of microvascular diseases like diabetes and renal failure (30). Consequently, the aggregate impact of these psychosocial and structural challenges can macroscopically obscure the genuinely protective associations of exercise facilities in unadjusted models.

In our fully adjusted models, we deliberately excluded obesity and diabetes prevalence from the covariate set because they act as mediators on the causal pathway of CKM syndrome rather than true baseline confounders, and exhibit strong collinearity with physical inactivity, which could otherwise induce over-adjustment bias. Instead, we included preventable hospital stay rates and food environment indices to capture healthcare access and nutritional contexts. Furthermore, while the residual Moran’s I indicated significant spatial clustering, our spatial error model sensitivity analysis yielded results consistent with the primary quasi Poisson models, reinforcing the reliability of our findings.

The inverse association between high exercise accessibility and CKM-related mortality possesses a robust epidemiological and physiological foundation (31–34). Regular physical activity (including leisure-time sports and active commuting) targets metabolic dysfunction, providing benefits to the cardiovascular system and the microvasculature-dense renal system by improving skeletal muscle insulin sensitivity, reducing visceral fat accumulation, and downregulating systemic chronic inflammation (35). Beyond direct biological benefits, exposure to outdoor green spaces such as urban parks can alleviate cognitive fatigue and psychological stress, reducing the allostatic load that drives cardiometabolic pathogenesis via neuroendocrine pathways, thereby delaying progressive target organ damage (36–38).

Furthermore, demographic and spatial stratification analyses revealed heterogeneity in these inverse associations across distinct subgroups. On one hand, the inverse association of ATEO attenuated with advancing age (with a stronger inverse association observed in the 45–64 age group compared to the ≥65 age group). This may be related to long-term, cumulative, and irreversible microvascular damage, suggesting that improving the exercise environment during early-to-mid adulthood may yield higher preventive potential. On the other hand, these associations exhibited significant urban–rural disparities: while ATEO demonstrated a significant inverse association with mortality in large and small metropolitan areas, this association diminished in noncore rural areas. This spatial heterogeneity indicates that merely increasing physical exercise infrastructure in rural settings may be insufficient to comprehensively improve health outcomes. Structural barriers prevalent in rural environments—such as low healthcare accessibility, long commuting distances, and a lack of complementary infrastructure (39, 40)—may restrict the capacity of vulnerable populations to translate physical activity opportunities into tangible health benefits.

Although this study reveals a tight correlation between the macro-exercise environment and CKM mortality, it faces certain limitations inherent to its ecological design. First, because this study relies entirely on county-level aggregated data, associations observed at the county level cannot be directly inferred as individual-level risk. Consequently, these findings represent macro-level associations rather than causal relationships. Second, our cross-sectional study design is based on data from 2014 to 2019. Since CKM mortality is a long-term outcome, current environmental indicators may not fully reflect the historical exposures leading to this mortality rate; therefore, our findings primarily reflect concurrent community characteristics rather than a causal timeline. Third, while we incorporated macro-level indicators such as the Food Environment Index and Preventable Hospital Stay rate into our fully adjusted models (Model 2), our analysis still lacked granular data on certain critical unmeasured confounders. Specifically, these aggregate county-level metrics cannot fully capture micro-level community vulnerabilities—such as household food insecurity—nor do they reflect the direct accessibility of cardiometabolic specialist healthcare, both of which are independent determinants of CKM mortality. Fourth, the current exposure metrics are relatively macroscopic and highly susceptible to spatial heterogeneity. Specifically, a single county-level ATEO average may mask extreme intra-county disparities, particularly in sprawling rural counties compared to dense urban ones. This unevenly distributed spatial noise likely contributed to the substantial attenuation of ATEO’s effect sizes following confounder adjustment. Additionally, these metrics fail to comprehensively capture micro-environmental barriers (e.g., facility usage fees, street safety) or occupational physical activity, potentially leading to measurement bias in exposure levels. Finally, other complex environmental elements characteristic of high-exercise-access areas might antagonize the health benefits of physical activity, such as heightened urban psychosocial stress or exposure to an “obesogenic environment” (12, 15, 41), and our reliance on aggregated data restricts the ability to incorporate precise individual follow-up times or competing risks models. Furthermore, county-level aggregation represents the finest spatial resolution publicly available via the CDC WONDER database, which objectively restricts our ability to analyze granular, intra-county neighborhood disparities.

## Conclusion

In summary, this nationwide ecological study provides evidence that county-level access to exercise opportunities is inversely associated with the mortality burden of overall CKM syndrome (Q4 vs. Q1, RR = 0.89) and its individual disease subtypes. Simultaneously, higher physical inactivity is associated with a significantly elevated CKM mortality risk (Q4 vs. Q1 RR = 1.23), predominantly driven by cardiovascular disease, though its associations with diabetes and renal failure were more heterogeneous. Reducing physical inactivity at the population level is therefore equally imperative. Favorable macro-built environments not only confer cardiovascular protection but also show consistent inverse associations with diabetes and renal failure mortality after full adjustment. These findings suggest that future public health strategies should transcend individual-level behavioral guidelines and pivot toward intersectoral macro-policy synergy. Deeply integrating the development of supportive exercise environments with community-based physical activity promotion, comprehensive chronic disease management, and healthy dietary interventions is essential to systemically alleviate the structural burden of CKM syndrome in the context of population aging and to substantially advance health equity.

## Data Availability

The datasets analyzed for this study can be found in publicly accessible repositories. Mortality data are available from the Centers for Disease Control and Prevention’s Wide-ranging Online Data for Epidemiologic Research (CDC WONDER) at https://wonder.cdc.gov/. County-level physical activity measures and the corresponding data documentation are available from the County Health Rankings & Roadmaps program (https://www.countyhealthrankings.org/health-data/methodology-and-sources/data-documentation). Both datasets are publicly accessible, and no specific permission was required for their use in this study.

## Footnotes

1
https://wonder.cdc.gov/

2
https://www.countyhealthrankings.org/health-data/methodology-and-sources/data-documentation

## Supplementary material

SUPPLEMENTARY FIGURE S1 Rate ratios and 95% confidence intervals for the association of access to exercise opportunities and physical inactivity with age-adjusted mortality from specific CKM syndrome components, stratified by sex. Estimates compare counties in higher quartiles (Q2, Q3, and Q4) to those in the lowest (Q1) quartile as the reference. Model 1 was adjusted for year. Model 2 was additionally adjusted for the same covariates as in Figure 2.

SUPPLEMENTARY FIGURE S2 Rate ratios and 95% confidence intervals for the association of access to exercise opportunities and physical inactivity with age-adjusted mortality from specific CKM syndrome components, stratified by urbanization level. Estimates compare counties in higher quartiles (Q2, Q3, and Q4) to those in the lowest (Q1) quartile as the reference. Model 1 was adjusted for year. Model 2 was additionally adjusted for the same covariates as in Figure 2.

SUPPLEMENTARY FIGURE S3 Rate ratios and 95% confidence intervals for the association of access to exercise opportunities and physical inactivity with age-adjusted mortality from specific CKM syndrome components, stratified by racial group. Estimates compare counties in higher quartiles (Q2, Q3, and Q4) to those in the lowest (Q1) quartile as the reference. Model 1 was adjusted for year. Model 2 was additionally adjusted for the same covariates as in Figure 2.

SUPPLEMENTARY FIGURE S4 Rate ratios and 95% confidence intervals for the association of access to exercise opportunities and physical inactivity with age-adjusted mortality from specific cardiovascular disease subtypes. Estimates compare counties in higher quartiles (Q2, Q3, and Q4) to those in the lowest (Q1) quartile as the reference. Model 1 was adjusted for year. Model 2 was additionally adjusted for the same covariates as in Figure 2.

SUPPLEMENTARY FIGURE S5 Dose–response relationship between access to exercise opportunities (ATEO) and CKM mortality derived from restricted cubic spline regression. The solid line represents the rate ratio relative to the median ATEO value (reference), with shaded areas indicating 95% confidence intervals. The model was adjusted for the same set of covariates as Model 2.

SUPPLEMENTARY FIGURE S6 Dose–response relationship between physical inactivity (PI) and CKM mortality derived from restricted cubic spline regression. The solid line represents the rate ratio relative to the median PI value (reference), with shaded areas indicating 95% confidence intervals. The model was adjusted for the same set of covariates as Model 2.

SUPPLEMENTARY Table S1 Age-Adjusted Mortality Rates for Specific Cardiovascular Disease Subtypes, by Quartile of County-Level Access to Exercise Opportunities and Physical Inactivity, US Counties, 2014–2019.

SUPPLEMENTARY Table S2 Percentage of Counties in the Extreme Quartiles of Each Indicator by U.S. Census Region, 2014 and 2019.

SUPPLEMENTARY Table S3 Model diagnostics for negative binomial regression models with clustered standard errors and spatial sensitivity analysis.
