# Supplementary material for: Associations of access to exercise opportunities and physical inactivity with cardiovascular–kidney–metabolic mortality: a nationwide ecological study
Source: Front Public Health. 2026 Oct 1;14:1924014. doi: 10.3389/fpubh.2026.1924014 (PMC13668831; doi:10.3389/fpubh.2026.1924014)
Supplement: Supplementary file 1 [file Data_Sheet_1.PDF]

(A) Male

ATEO PI

Disease	Model	Series	Q2 vs Q1	P value	Q3 vs Q1	P value	Q4 vs Q1	P value
Cardio−Kidney−Metabolic diseases	Model 1	ATEO	0.89(0.87,0.91)	<0.0001	0.81(0.79,0.82)	<0.0001	0.65(0.64,0.66)	<0.0001
		PI	1.28(1.27,1.29)	<0.0001	1.47(1.46,1.49)	<0.0001	1.70(1.68,1.73)	<0.0001
	Model 2	ATEO	0.98(0.96,0.99)	0.004	0.95(0.93,0.96)	<0.0001	0.90(0.89,0.92)	<0.0001
		PI	1.10(1.09,1.10)	<0.0001	1.16(1.15,1.17)	<0.0001	1.20(1.19,1.22)	<0.0001
Cardiovascular disease	Model 1	ATEO	0.89(0.87,0.91)	<0.0001	0.81(0.79,0.83)	<0.0001	0.65(0.64,0.66)	<0.0001
		PI	1.27(1.26,1.29)	<0.0001	1.47(1.45,1.49)	<0.0001	1.70(1.67,1.72)	<0.0001
	Model 2	ATEO	0.98(0.97,1.00)	0.02	0.95(0.93,0.96)	<0.0001	0.90(0.89,0.92)	<0.0001
		PI	1.10(1.09,1.11)	<0.0001	1.17(1.15,1.18)	<0.0001	1.21(1.20,1.23)	<0.0001
Diabetes mellitus	Model 1	ATEO	0.89(0.83,0.95)	<0.001	0.78(0.74,0.83)	<0.0001	0.58(0.55,0.61)	<0.0001
		PI	1.22(1.20,1.25)	<0.0001	1.49(1.45,1.54)	<0.0001	1.90(1.83,1.98)	<0.0001
	Model 2	ATEO	1.01(0.94,1.07)	0.84	0.98(0.92,1.04)	0.54	0.93(0.87,0.99)	0.03
		PI	1.03(1.01,1.05)	0.01	1.12(1.09,1.16)	<0.0001	1.22(1.17,1.27)	<0.0001
Renal failure	Model 1	ATEO	1.36(1.21,1.52)	<0.0001	1.41(1.26,1.57)	<0.0001	1.03(0.93,1.15)	0.54
		PI	1.41(1.37,1.45)	<0.0001	1.50(1.44,1.55)	<0.0001	1.64(1.55,1.74)	<0.0001
	Model 2	ATEO	1.45(1.28,1.65)	<0.0001	1.37(1.21,1.55)	<0.0001	1.13(0.99,1.28)	0.07
		PI	1.08(1.05,1.11)	<0.0001	1.05(1.01,1.10)	0.03	1.06(0.99,1.14)	0.08

(B) Female

ATEO PI

Disease	Model	Series	Q2 vs Q1	P value	Q3 vs Q1	P value	Q4 vs Q1	P value
Cardio−Kidney−Metabolic diseases	Model 1	ATEO	0.89(0.87,0.91)	<0.0001	0.81(0.80,0.83)	<0.0001	0.66(0.65,0.67)	<0.0001
		PI	1.27(1.26,1.29)	<0.0001	1.46(1.45,1.48)	<0.0001	1.67(1.64,1.69)	<0.0001
	Model 2	ATEO	0.97(0.95,0.98)	<0.001	0.92(0.91,0.94)	<0.0001	0.87(0.86,0.89)	<0.0001
		PI	1.13(1.12,1.13)	<0.0001	1.21(1.19,1.22)	<0.0001	1.26(1.24,1.28)	<0.0001
Cardiovascular disease	Model 1	ATEO	0.89(0.87,0.92)	<0.0001	0.82(0.80,0.84)	<0.0001	0.68(0.66,0.69)	<0.0001
		PI	1.26(1.25,1.28)	<0.0001	1.44(1.42,1.46)	<0.0001	1.63(1.61,1.66)	<0.0001
	Model 2	ATEO	0.97(0.95,0.98)	<0.001	0.92(0.91,0.94)	<0.0001	0.88(0.86,0.90)	<0.0001
		PI	1.13(1.12,1.14)	<0.0001	1.21(1.20,1.22)	<0.0001	1.27(1.25,1.29)	<0.0001
Diabetes mellitus	Model 1	ATEO	0.98(0.91,1.06)	0.62	0.85(0.79,0.91)	<0.0001	0.61(0.57,0.65)	<0.0001
		PI	1.25(1.23,1.28)	<0.0001	1.59(1.54,1.63)	<0.0001	2.06(1.98,2.15)	<0.0001
	Model 2	ATEO	1.12(1.04,1.22)	0.004	1.07(0.99,1.15)	0.10	0.95(0.88,1.03)	0.25
		PI	1.06(1.03,1.09)	<0.0001	1.23(1.18,1.27)	<0.0001	1.35(1.29,1.42)	<0.0001
Renal failure	Model 1	ATEO	1.28(1.14,1.44)	<0.0001	1.30(1.16,1.45)	<0.0001	0.91(0.82,1.02)	0.09
		PI	1.44(1.40,1.48)	<0.0001	1.60(1.55,1.66)	<0.0001	1.81(1.71,1.92)	<0.0001
	Model 2	ATEO	1.56(1.36,1.78)	<0.0001	1.41(1.24,1.61)	<0.0001	1.16(1.01,1.33)	0.03
		PI	1.09(1.06,1.13)	<0.0001	1.10(1.05,1.15)	<0.0001	1.11(1.04,1.20)	0.003
