# Supplementary material for: Associations of access to exercise opportunities and physical inactivity with cardiovascular–kidney–metabolic mortality: a nationwide ecological study
Source: Front Public Health. 2026 Oct 1;14:1924014. doi: 10.3389/fpubh.2026.1924014 (PMC13668831; doi:10.3389/fpubh.2026.1924014)
Supplement: Supplementary file 2 [file Data_Sheet_2.PDF]

(A) Large Fringe Metro

ATEO PI

Disease	Model	Q2 vs Q1	P value	Q3 vs Q1	P value	Q4 vs Q1	P value
Cardio−Kidney−Metabolic diseases	Model 1	0.95(0.87,1.05)	0.33	0.80(0.73,0.87)	<0.0001	0.77(0.71,0.83)	<0.0001
		1.25(1.22,1.28)	<0.0001	1.35(1.30,1.40)	<0.0001	1.46(1.37,1.56)	<0.0001
	Model 2	0.99(0.93,1.06)	0.87	0.90(0.84,0.96)	<0.001	0.88(0.82,0.94)	<0.001
		1.08(1.06,1.10)	<0.0001	1.09(1.06,1.12)	<0.0001	1.17(1.12,1.23)	<0.0001
Cardiovascular disease	Model 1	0.96(0.87,1.06)	0.45	0.80(0.73,0.87)	<0.0001	0.78(0.71,0.84)	<0.0001
		1.24(1.21,1.28)	<0.0001	1.32(1.27,1.38)	<0.0001	1.44(1.35,1.54)	<0.0001
	Model 2	1.00(0.94,1.07)	0.93	0.90(0.84,0.96)	0.002	0.89(0.83,0.95)	<0.001
		1.08(1.06,1.10)	<0.0001	1.08(1.05,1.12)	<0.0001	1.18(1.12,1.24)	<0.0001
Diabetes mellitus	Model 1	0.80(0.68,0.95)	0.01	0.69(0.59,0.80)	<0.0001	0.58(0.50,0.67)	<0.0001
		1.23(1.18,1.28)	<0.0001	1.58(1.49,1.67)	<0.0001	1.81(1.64,2.00)	<0.0001
	Model 2	0.82(0.70,0.95)	0.01	0.78(0.68,0.90)	<0.001	0.77(0.66,0.90)	<0.001
		1.01(0.97,1.05)	0.71	1.11(1.05,1.18)	<0.001	1.18(1.07,1.31)	0.001
Renal failure	Model 1	0.97(0.77,1.22)	0.78	0.79(0.64,0.98)	0.03	0.70(0.57,0.86)	<0.001
		1.32(1.26,1.38)	<0.0001	1.60(1.51,1.71)	<0.0001	1.76(1.55,2.00)	<0.0001
	Model 2	0.99(0.77,1.28)	0.96	0.85(0.67,1.09)	0.21	0.79(0.61,1.01)	0.06
		1.11(1.07,1.17)	<0.0001	1.19(1.10,1.28)	<0.0001	1.27(1.11,1.44)	<0.001

(B) Medium Metro

ATEO PI

Disease	Model	Q2 vs Q1	P value	Q3 vs Q1	P value	Q4 vs Q1	P value
Cardio−Kidney−Metabolic diseases	Model 1	0.84(0.79,0.90)	<0.0001	0.85(0.80,0.91)	<0.0001	0.78(0.73,0.83)	<0.0001
		1.27(1.24,1.29)	<0.0001	1.41(1.37,1.44)	<0.0001	1.52(1.46,1.58)	<0.0001
	Model 2	0.98(0.93,1.03)	0.43	0.98(0.94,1.03)	0.51	0.96(0.91,1.01)	0.15
		1.14(1.12,1.15)	<0.0001	1.21(1.18,1.24)	<0.0001	1.25(1.21,1.29)	<0.0001
Cardiovascular disease	Model 1	0.84(0.78,0.90)	<0.0001	0.86(0.80,0.91)	<0.0001	0.79(0.74,0.84)	<0.0001
		1.26(1.24,1.29)	<0.0001	1.40(1.36,1.44)	<0.0001	1.50(1.44,1.57)	<0.0001
	Model 2	0.98(0.93,1.04)	0.55	0.99(0.94,1.05)	0.74	0.97(0.91,1.02)	0.24
		1.14(1.12,1.16)	<0.0001	1.22(1.19,1.25)	<0.0001	1.26(1.22,1.31)	<0.0001
Diabetes mellitus	Model 1	0.81(0.71,0.91)	<0.001	0.76(0.68,0.85)	<0.0001	0.68(0.61,0.76)	<0.0001
		1.21(1.17,1.26)	<0.0001	1.41(1.35,1.48)	<0.0001	1.66(1.55,1.78)	<0.0001
	Model 2	0.89(0.78,1.02)	0.10	0.91(0.80,1.03)	0.14	0.96(0.84,1.10)	0.57
		1.04(1.00,1.08)	0.06	1.12(1.06,1.19)	<0.0001	1.21(1.11,1.32)	<0.0001
Renal failure	Model 1	0.77(0.65,0.90)	0.002	0.74(0.64,0.87)	<0.001	0.62(0.53,0.72)	<0.0001
		1.48(1.42,1.55)	<0.0001	1.60(1.51,1.69)	<0.0001	1.92(1.76,2.09)	<0.0001
	Model 2	0.91(0.76,1.08)	0.27	0.80(0.67,0.95)	0.01	0.74(0.62,0.88)	<0.001
		1.23(1.17,1.30)	<0.0001	1.20(1.12,1.29)	<0.0001	1.28(1.15,1.43)	<0.0001

(C)Micropolitan (Nonmetro)

AETO PI

Disease	Model	Q2 vs Q1	P value	Q3 vs Q1	P value	Q4 vs Q1	P value
Cardio−Kidney−Metabolic diseases	Model 1	0.99(0.96,1.02)	0.48	0.95(0.92,0.97)	<0.0001	0.83(0.81,0.86)	<0.0001
		1.13(1.11,1.16)	<0.0001	1.27(1.25,1.30)	<0.0001	1.40(1.37,1.43)	<0.0001
	Model 2	0.97(0.94,0.99)	0.01	0.95(0.93,0.97)	<0.0001	0.90(0.87,0.93)	<0.0001
		1.08(1.06,1.10)	<0.0001	1.13(1.10,1.15)	<0.0001	1.18(1.15,1.21)	<0.0001
Cardiovascular disease	Model 1	1.00(0.97,1.03)	0.94	0.96(0.93,0.99)	0.004	0.85(0.82,0.88)	<0.0001
		1.11(1.09,1.14)	<0.0001	1.24(1.22,1.27)	<0.0001	1.37(1.34,1.40)	<0.0001
	Model 2	0.99(0.96,1.01)	0.25	0.97(0.94,0.99)	0.01	0.92(0.89,0.94)	<0.0001
		1.08(1.06,1.10)	<0.0001	1.14(1.11,1.16)	<0.0001	1.18(1.15,1.22)	<0.0001
Diabetes mellitus	Model 1	0.93(0.87,0.98)	0.01	0.87(0.82,0.92)	<0.0001	0.76(0.71,0.81)	<0.0001
		1.19(1.14,1.25)	<0.0001	1.30(1.25,1.37)	<0.0001	1.47(1.41,1.54)	<0.0001
	Model 2	0.92(0.86,0.97)	0.005	0.91(0.85,0.96)	0.002	0.90(0.84,0.96)	0.003
		1.04(0.99,1.09)	0.14	1.03(0.98,1.09)	0.24	1.07(1.01,1.14)	0.03
Renal failure	Model 1	0.95(0.88,1.01)	0.11	0.89(0.83,0.95)	<0.001	0.77(0.71,0.83)	<0.0001
		1.36(1.28,1.44)	<0.0001	1.54(1.45,1.62)	<0.0001	1.63(1.54,1.73)	<0.0001
	Model 2	0.93(0.87,1.00)	0.05	0.94(0.88,1.02)	0.13	0.93(0.85,1.01)	0.09
		1.20(1.13,1.28)	<0.0001	1.28(1.19,1.37)	<0.0001	1.29(1.20,1.40)	<0.0001

(D) NonCore (Nonmetro)

ATEO PI

Disease	Model	Q2 vs Q1	P value	Q3 vs Q1	P value	Q4 vs Q1	P value
Cardio−Kidney−Metabolic diseases	Model 1	0.98(0.96,0.99)	<0.001	0.93(0.92,0.95)	<0.0001	0.92(0.90,0.94)	<0.0001
		1.14(1.12,1.15)	<0.0001	1.22(1.20,1.25)	<0.0001	1.30(1.28,1.32)	<0.0001
	Model 2	0.98(0.97,0.99)	0.004	0.94(0.92,0.95)	<0.0001	0.89(0.87,0.90)	<0.0001
		1.15(1.13,1.17)	<0.0001	1.23(1.21,1.25)	<0.0001	1.30(1.28,1.32)	<0.0001
Cardiovascular disease	Model 1	0.98(0.97,1.00)	0.02	0.95(0.93,0.96)	<0.0001	0.93(0.91,0.95)	<0.0001
		1.13(1.11,1.15)	<0.0001	1.21(1.19,1.24)	<0.0001	1.28(1.26,1.31)	<0.0001
	Model 2	0.98(0.97,1.00)	0.02	0.94(0.93,0.96)	<0.0001	0.89(0.87,0.91)	<0.0001
		1.15(1.13,1.17)	<0.0001	1.23(1.21,1.25)	<0.0001	1.30(1.27,1.33)	<0.0001
Diabetes mellitus	Model 1	0.92(0.87,0.96)	<0.001	0.85(0.80,0.90)	<0.0001	0.85(0.77,0.93)	<0.001
		1.12(1.05,1.20)	0.001	1.20(1.12,1.29)	<0.0001	1.45(1.36,1.54)	<0.0001
	Model 2	0.94(0.88,0.99)	0.02	0.89(0.83,0.96)	0.001	0.95(0.86,1.06)	0.36
		1.10(1.02,1.19)	0.02	1.12(1.04,1.22)	0.003	1.24(1.15,1.35)	<0.0001
Renal failure	Model 1	1.17(1.07,1.27)	<0.001	0.89(0.80,0.99)	0.03	0.61(0.51,0.73)	<0.0001
		1.61(1.39,1.86)	<0.0001	2.10(1.83,2.40)	<0.0001	2.70(2.37,3.09)	<0.0001
	Model 2	1.07(0.97,1.17)	0.20	0.88(0.78,1.00)	0.05	0.70(0.58,0.85)	<0.001
		1.40(1.21,1.63)	<0.0001	1.66(1.43,1.93)	<0.0001	1.81(1.54,2.12)	<0.0001

(E) Small Metro

ATEO PI

Disease	Model	Q2 vs Q1	P value	Q3 vs Q1	P value	Q4 vs Q1	P value
Cardio−Kidney−Metabolic diseases	Model 1	0.91(0.86,0.96)	<0.001	0.88(0.84,0.93)	<0.0001	0.80(0.76,0.84)	<0.0001
		1.21(1.17,1.24)	<0.0001	1.39(1.35,1.44)	<0.0001	1.50(1.45,1.56)	<0.0001
	Model 2	0.95(0.91,1.00)	0.03	0.97(0.93,1.01)	0.13	0.92(0.88,0.97)	<0.001
		1.14(1.11,1.16)	<0.0001	1.26(1.23,1.29)	<0.0001	1.29(1.25,1.33)	<0.0001
Cardiovascular disease	Model 1	0.90(0.85,0.96)	0.001	0.89(0.84,0.93)	<0.0001	0.81(0.77,0.86)	<0.0001
		1.19(1.16,1.23)	<0.0001	1.37(1.33,1.42)	<0.0001	1.49(1.43,1.55)	<0.0001
	Model 2	0.96(0.91,1.00)	0.06	0.97(0.93,1.02)	0.19	0.93(0.89,0.98)	0.005
		1.13(1.10,1.16)	<0.0001	1.25(1.22,1.29)	<0.0001	1.30(1.25,1.34)	<0.0001
Diabetes mellitus	Model 1	0.86(0.76,0.97)	0.01	0.81(0.73,0.91)	<0.001	0.69(0.62,0.77)	<0.0001
		1.20(1.13,1.27)	<0.0001	1.44(1.36,1.53)	<0.0001	1.48(1.37,1.60)	<0.0001
	Model 2	0.86(0.75,0.97)	0.02	0.89(0.79,1.00)	0.06	0.84(0.74,0.96)	0.01
		1.09(1.02,1.16)	0.01	1.23(1.15,1.32)	<0.0001	1.16(1.05,1.28)	0.003
Renal failure	Model 1	0.99(0.87,1.13)	0.93	0.94(0.83,1.07)	0.35	0.81(0.72,0.92)	0.001
		1.34(1.26,1.42)	<0.0001	1.55(1.46,1.65)	<0.0001	1.55(1.44,1.68)	<0.0001
	Model 2	1.01(0.87,1.17)	0.90	1.02(0.88,1.17)	0.83	0.92(0.80,1.07)	0.30
		1.24(1.16,1.32)	<0.0001	1.35(1.25,1.45)	<0.0001	1.27(1.15,1.40)	<0.0001
