# Supplementary material for: Associations of access to exercise opportunities and physical inactivity with cardiovascular–kidney–metabolic mortality: a nationwide ecological study
Source: Front Public Health. 2026 Oct 1;14:1924014. doi: 10.3389/fpubh.2026.1924014 (PMC13668831; doi:10.3389/fpubh.2026.1924014)
Supplement: Supplementary file 3 [file Data_Sheet_3.PDF]

(A) American Indian or Alaska Native

ATEO PI

Disease	Model	Q2 vs Q1	P value	Q3 vs Q1	P value	Q4 vs Q1	P value
Cardio−Kidney−Metabolic diseases	Model 1	0.84(0.81,0.87)	<0.0001	0.58(0.55,0.60)	<0.0001	0.45(0.44,0.47)	<0.0001
		1.39(1.34,1.43)	<0.0001	1.52(1.47,1.58)	<0.0001	2.20(2.12,2.28)	<0.0001
	Model 2	1.07(1.02,1.11)	0.004	0.90(0.85,0.95)	<0.0001	0.93(0.88,0.98)	0.01
		0.86(0.83,0.89)	<0.0001	0.86(0.82,0.90)	<0.0001	1.05(1.00,1.10)	0.05
Cardiovascular disease	Model 1	0.84(0.80,0.87)	<0.0001	0.55(0.52,0.58)	<0.0001	0.48(0.47,0.50)	<0.0001
		1.29(1.24,1.33)	<0.0001	1.49(1.43,1.55)	<0.0001	2.30(2.22,2.39)	<0.0001
	Model 2	1.10(1.05,1.15)	<0.001	0.86(0.81,0.92)	<0.0001	0.98(0.92,1.04)	0.43
		0.81(0.77,0.84)	<0.0001	0.85(0.81,0.90)	<0.0001	1.16(1.10,1.23)	<0.0001
Diabetes mellitus	Model 1	0.56(0.52,0.60)	<0.0001	0.23(0.21,0.25)	<0.0001	0.22(0.20,0.23)	<0.0001
		1.45(1.37,1.54)	<0.0001	0.65(0.59,0.73)	<0.0001	1.17(1.06,1.29)	0.002
	Model 2	0.74(0.68,0.82)	<0.0001	0.44(0.38,0.50)	<0.0001	0.42(0.37,0.48)	<0.0001
		0.88(0.83,0.94)	<0.001	0.65(0.58,0.72)	<0.0001	0.78(0.69,0.88)	<0.0001

0 0.5 1 1.5 2

(B) Black or African American

ATEO PI

Disease	Model	Q2 vs Q1	P value	Q3 vs Q1	P value	Q4 vs Q1	P value
Cardio−Kidney−Metabolic diseases	Model 1	0.85(0.83,0.88)	<0.0001	0.71(0.69,0.73)	<0.0001	0.65(0.63,0.66)	<0.0001
		1.18(1.16,1.19)	<0.0001	1.36(1.34,1.38)	<0.0001	1.55(1.52,1.58)	<0.0001
	Model 2	0.94(0.91,0.97)	<0.001	0.85(0.83,0.88)	<0.0001	0.84(0.81,0.87)	<0.0001
		1.02(1.00,1.03)	0.01	1.07(1.05,1.09)	<0.0001	1.06(1.03,1.08)	<0.0001
Cardiovascular disease	Model 1	0.85(0.82,0.87)	<0.0001	0.71(0.69,0.73)	<0.0001	0.66(0.65,0.68)	<0.0001
		1.18(1.16,1.19)	<0.0001	1.35(1.33,1.38)	<0.0001	1.54(1.51,1.57)	<0.0001
	Model 2	0.93(0.89,0.96)	<0.0001	0.83(0.80,0.87)	<0.0001	0.82(0.78,0.85)	<0.0001
		1.03(1.01,1.05)	<0.001	1.08(1.06,1.11)	<0.0001	1.08(1.05,1.11)	<0.0001
Diabetes mellitus	Model 1	0.86(0.80,0.91)	<0.0001	0.76(0.72,0.80)	<0.0001	0.67(0.63,0.70)	<0.0001
		1.12(1.10,1.14)	<0.0001	1.32(1.29,1.35)	<0.0001	1.60(1.55,1.65)	<0.0001
	Model 2	0.91(0.84,0.97)	0.01	0.78(0.73,0.84)	<0.0001	0.74(0.68,0.80)	<0.0001
		0.96(0.94,0.98)	<0.001	1.07(1.04,1.10)	<0.0001	1.13(1.08,1.18)	<0.0001
Renal failure	Model 1	1.48(1.34,1.64)	<0.0001	1.49(1.35,1.63)	<0.0001	1.43(1.31,1.56)	<0.0001
		1.13(1.11,1.15)	<0.0001	1.09(1.06,1.12)	<0.0001	1.02(0.98,1.07)	0.36
	Model 2	1.17(1.04,1.33)	0.01	0.96(0.85,1.09)	0.56	0.91(0.80,1.04)	0.16
		0.86(0.83,0.88)	<0.0001	0.82(0.79,0.85)	<0.0001	0.80(0.75,0.85)	<0.0001

0 0.5 1 1.5 2

(C)White

AETO PI

Disease	Model	Q2 vs Q1	P value	Q3 vs Q1	P value	Q4 vs Q1	P value
Cardio−Kidney−Metabolic diseases	Model 1	0.90(0.88,0.91)	<0.0001	0.83(0.81,0.84)	<0.0001	0.68(0.67,0.69)	<0.0001
		1.28(1.26,1.29)	<0.0001	1.46(1.45,1.48)	<0.0001	1.65(1.63,1.67)	<0.0001
	Model 2	0.98(0.97,1.00)	0.01	0.96(0.95,0.98)	<0.0001	0.93(0.91,0.94)	<0.0001
		1.09(1.08,1.10)	<0.0001	1.15(1.14,1.16)	<0.0001	1.17(1.16,1.19)	<0.0001
Cardiovascular disease	Model 1	0.89(0.88,0.91)	<0.0001	0.83(0.81,0.85)	<0.0001	0.69(0.68,0.71)	<0.0001
		1.27(1.26,1.28)	<0.0001	1.45(1.44,1.47)	<0.0001	1.63(1.61,1.66)	<0.0001
	Model 2	0.98(0.96,0.99)	0.002	0.96(0.95,0.98)	<0.0001	0.92(0.91,0.93)	<0.0001
		1.08(1.07,1.09)	<0.0001	1.15(1.14,1.17)	<0.0001	1.19(1.17,1.20)	<0.0001
Diabetes mellitus	Model 1	0.88(0.84,0.92)	<0.0001	0.78(0.74,0.81)	<0.0001	0.57(0.55,0.60)	<0.0001
		1.25(1.22,1.27)	<0.0001	1.53(1.49,1.56)	<0.0001	1.89(1.83,1.95)	<0.0001
	Model 2	0.93(0.89,0.97)	<0.001	0.92(0.89,0.96)	<0.001	0.89(0.85,0.93)	<0.0001
		1.03(1.01,1.05)	0.01	1.10(1.07,1.13)	<0.0001	1.15(1.11,1.19)	<0.0001
Renal failure	Model 1	0.96(0.89,1.03)	0.27	0.87(0.81,0.93)	<0.001	0.62(0.58,0.66)	<0.0001
		1.45(1.42,1.49)	<0.0001	1.66(1.61,1.71)	<0.0001	1.99(1.90,2.07)	<0.0001
	Model 2	0.96(0.90,1.02)	0.15	0.94(0.88,1.00)	0.05	0.85(0.80,0.91)	<0.0001
		1.11(1.08,1.14)	<0.0001	1.12(1.09,1.16)	<0.0001	1.14(1.09,1.20)	<0.0001

0 0.5 1 1.5 2
