# Supplementary material for: Associations of access to exercise opportunities and physical inactivity with cardiovascular–kidney–metabolic mortality: a nationwide ecological study
Source: Front Public Health. 2026 Oct 1;14:1924014. doi: 10.3389/fpubh.2026.1924014 (PMC13668831; doi:10.3389/fpubh.2026.1924014)
Supplement: Supplementary file 4 [file Data_Sheet_4.PDF]

ATEO PI

Disease	Model	Q2 vs Q1	P value	Q3 vs Q1	P value	Q4 vs Q1	P value
Acute myocardial infarction	Model 1	0.77(0.74,0.81)	<0.0001	0.59(0.57,0.62)	<0.0001	0.35(0.34,0.37)	<0.0001
		1.45(1.41,1.48)	<0.0001	1.92(1.86,1.98)	<0.0001	2.83(2.74,2.92)	<0.0001
	Model 2	0.96(0.91,1.00)	0.06	0.88(0.84,0.92)	<0.0001	0.77(0.73,0.81)	<0.0001
		1.18(1.15,1.20)	<0.0001	1.28(1.24,1.33)	<0.0001	1.47(1.41,1.53)	<0.0001
Atrial fibrillation	Model 1	1.73(1.51,1.99)	<0.0001	2.00(1.76,2.27)	<0.0001	1.46(1.29,1.66)	<0.0001
		1.07(1.04,1.10)	<0.0001	1.09(1.04,1.14)	<0.001	0.95(0.88,1.04)	0.29
	Model 2	2.14(1.84,2.48)	<0.0001	2.35(2.04,2.71)	<0.0001	2.09(1.81,2.42)	<0.0001
		0.94(0.91,0.97)	<0.0001	0.90(0.86,0.95)	<0.0001	0.74(0.68,0.81)	<0.0001
Cerebrovascular diseases	Model 1	0.88(0.86,0.91)	<0.0001	0.82(0.79,0.84)	<0.0001	0.68(0.66,0.70)	<0.0001
		1.15(1.14,1.17)	<0.0001	1.32(1.30,1.35)	<0.0001	1.51(1.48,1.54)	<0.0001
	Model 2	0.97(0.94,0.99)	0.02	0.93(0.90,0.96)	<0.0001	0.91(0.88,0.94)	<0.0001
		0.99(0.97,1.00)	0.05	1.04(1.02,1.06)	<0.0001	1.08(1.05,1.11)	<0.0001
Heart failure	Model 1	0.79(0.76,0.83)	<0.0001	0.70(0.67,0.73)	<0.0001	0.52(0.50,0.54)	<0.0001
		1.27(1.25,1.29)	<0.0001	1.50(1.46,1.53)	<0.0001	1.92(1.87,1.98)	<0.0001
	Model 2	0.92(0.88,0.96)	<0.0001	0.88(0.84,0.92)	<0.0001	0.82(0.78,0.86)	<0.0001
		1.06(1.04,1.08)	<0.0001	1.11(1.08,1.14)	<0.0001	1.20(1.16,1.24)	<0.0001
Ischemic heart disease	Model 1	0.90(0.88,0.93)	<0.0001	0.81(0.78,0.83)	<0.0001	0.66(0.64,0.68)	<0.0001
		1.33(1.31,1.34)	<0.0001	1.54(1.51,1.56)	<0.0001	1.75(1.72,1.79)	<0.0001
	Model 2	0.98(0.96,1.01)	0.21	0.94(0.91,0.96)	<0.0001	0.90(0.87,0.93)	<0.0001
		1.21(1.20,1.23)	<0.0001	1.33(1.31,1.35)	<0.0001	1.41(1.38,1.44)	<0.0001
