# Supplementary material for: Associations of access to exercise opportunities and physical inactivity with cardiovascular–kidney–metabolic mortality: a nationwide ecological study
Source: Front Public Health. 2026 Oct 1;14:1924014. doi: 10.3389/fpubh.2026.1924014 (PMC13668831; doi:10.3389/fpubh.2026.1924014)
Supplement: Supplementary file 5 [file Data_Sheet_5.PDF]

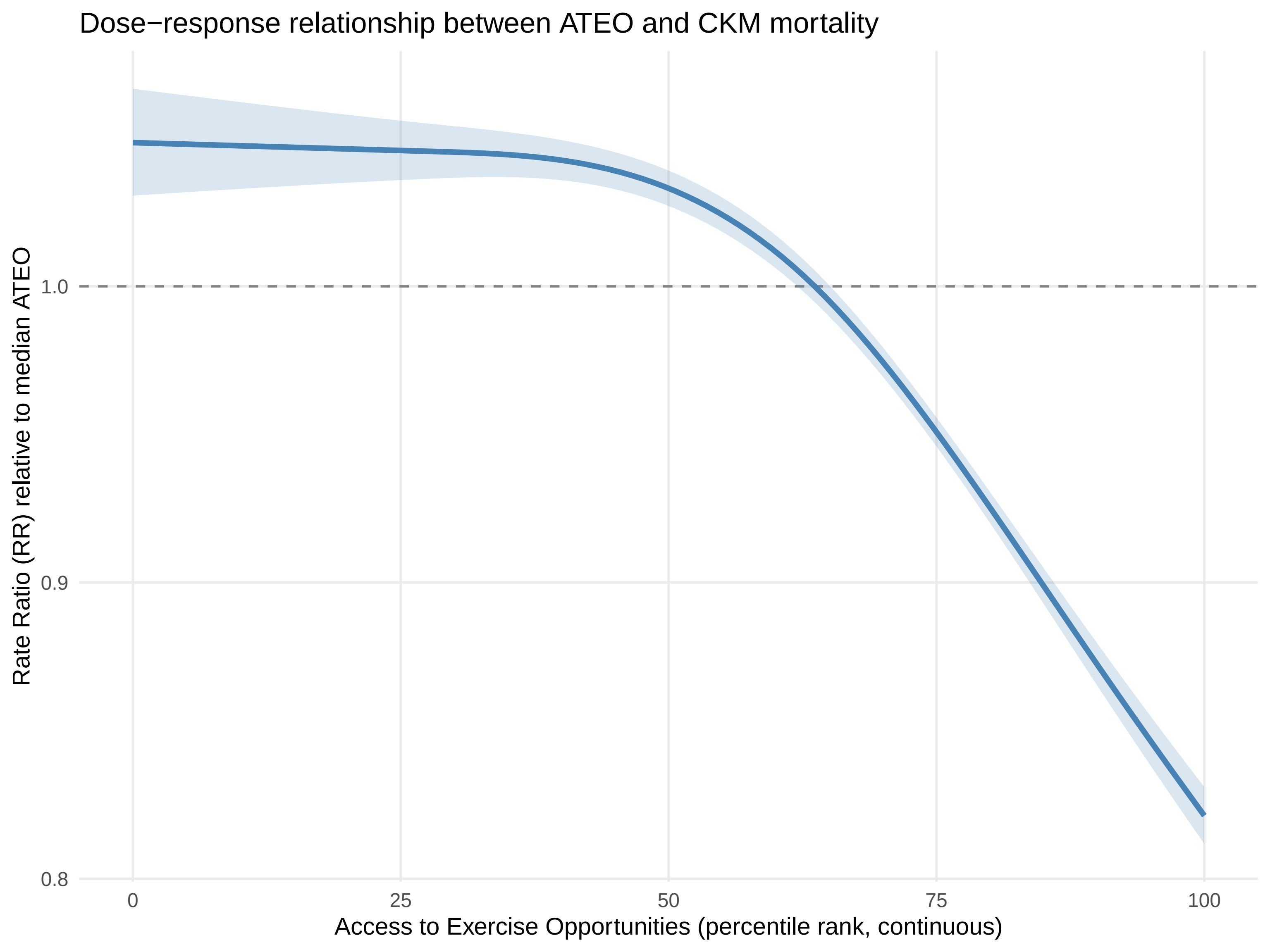
Dose−response relationship between ATEO and CKM mortality
Rate Ratio (RR) relative to median ATEO
1.0
0.9
0.8
0
25
50
75
100
Access to Exercise Opportunities (percentile rank, continuous)
