# Supplementary material for: Associations of access to exercise opportunities and physical inactivity with cardiovascular–kidney–metabolic mortality: a nationwide ecological study
Source: Front Public Health. 2026 Oct 1;14:1924014. doi: 10.3389/fpubh.2026.1924014 (PMC13668831; doi:10.3389/fpubh.2026.1924014)
Supplement: Supplementary file 6 [file Data_Sheet_6.PDF]

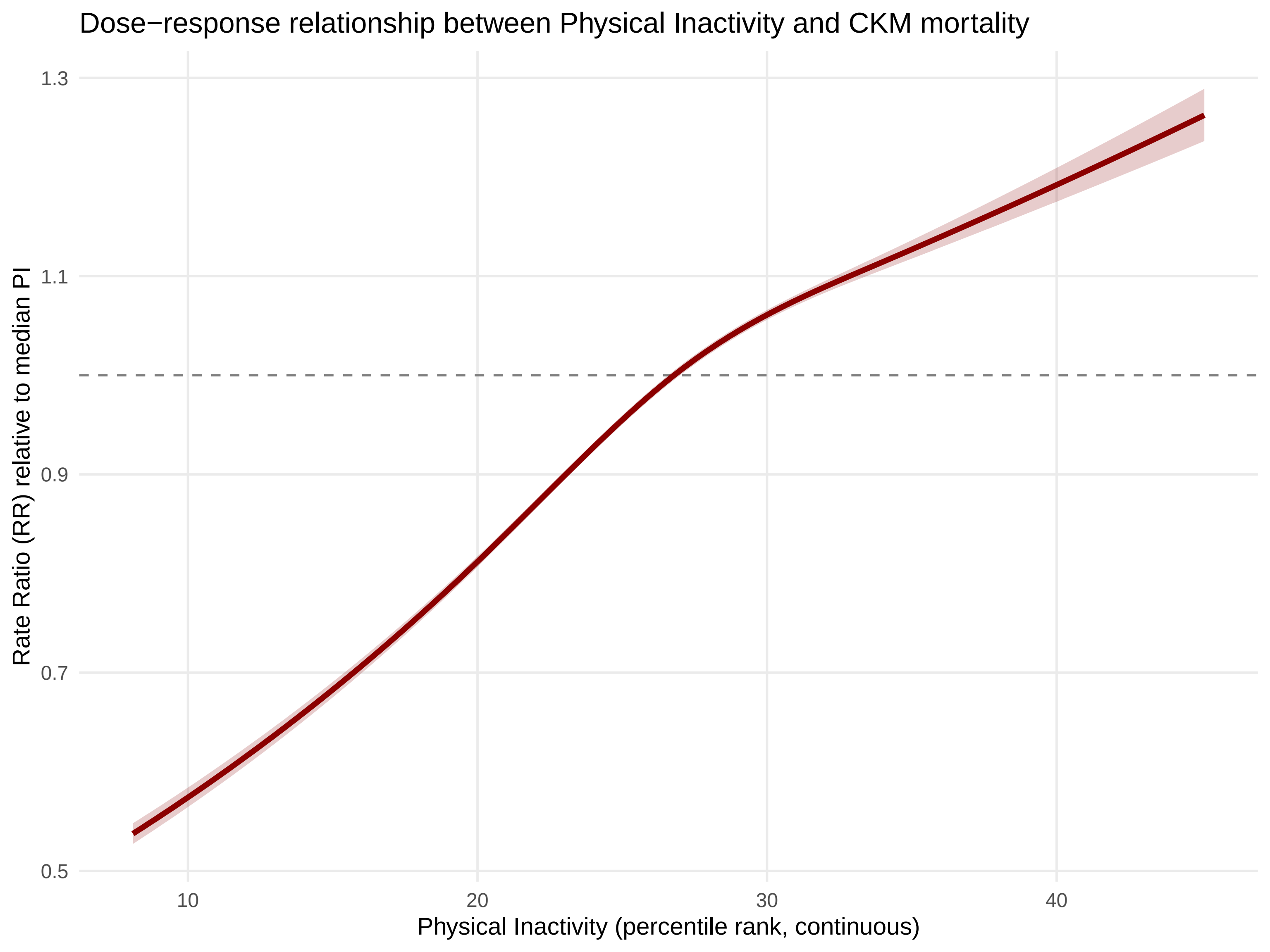
Dose−response relationship between Physical Inactivity and CKM mortality
Rate Ratio (RR) relative to median PI
1.3
1.1
0.9
0.7
0.5
10
20
30
40
Physical Inactivity (percentile rank, continuous)
