# Supplementary material for: Associations of access to exercise opportunities and physical inactivity with cardiovascular–kidney–metabolic mortality: a nationwide ecological study
Source: Front Public Health. 2026 Oct 1;14:1924014. doi: 10.3389/fpubh.2026.1924014 (PMC13668831; doi:10.3389/fpubh.2026.1924014)
Supplement: Supplementary file 7 [file Table_1.docx]

**Table S1**. Age-Adjusted Mortality Rates for Specific Cardiovascular Disease Subtypes, by Quartile of County-Level Access to Exercise Opportunities and Physical Inactivity, U.S. Counties, 2014–2019

| **Cardio-Kidney-Metabolic**  **mortality** | **Q1** | **Q2** | **Q3** | **Q4** | **p value for trend*** | **Rate ratio quartile 4/1‡** | **p value†** |
| --- | --- | --- | --- | --- | --- | --- | --- |
|  | **Access to exercise opportunities** | | | | | | |
| Acute myocardial infarction | 115.46(113.5, 117.42) | 132.43(131.11, 133.77) | 106.42(105.62, 107.23) | 66.17(65.94, 66.41) | <0.0001 | 0.77(0.73, 0.81) | <0.0001 |
| Atrial fibrillation | 1.85(1.64, 2.07) | 5.80(5.48, 6.12) | 13.31(12.97, 13.66) | 16.30(16.17, 16.43) | <0.0001 | 2.09(1.81, 2.42) | 0.16 |
| Cerebrovascular diseases | 90.75(89.01, 92.50) | 108.48(107.4, 109.58) | 110.42(109.7, 111.14) | 100.92(100.64, 101.2) | 0.004 | 0.91(0.88, 0.94) | <0.0001 |
| Heart failure | 75.2(73.62, 76.8) | 79.73(78.76, 80.71) | 82.48(81.83, 83.13) | 67.76(67.53, 67.99) | 0.005 | 0.82(0.78, 0.86) | <0.0001 |
| Ischemic heart disease | 333.86(331.32, 336.41) | 320.86(319.3, 322.43) | 288.56(287.52, 289.6) | 249.81(249.39, 250.23) | <0.0001 | 0.90(0.87, 0.93) | <0.0001 |
|  | **Physical inactivity** | | | | | | |
| Acute myocardial infarction | 62.31(62.05, 62.57) | 81.83(81.36, 82.3) | 98.64(97.87, 99.42) | 152.27(150.69, 153.86) | <0.0001 | 1.47(1.41, 1.53) | <0.0001 |
| Atrial fibrillation | 16.46(16.32, 16.61) | 14.68(14.46, 14.91) | 9.83(9.55, 10.11) | 2.86(2.63, 3.1) | <0.0001 | 0.74(0.68, 0.81) | 0.21 |
| Cerebrovascular diseases | 98.85(98.53, 99.16) | 104.77(104.26, 105.27) | 111.17(110.4, 111.95) | 115.33(114.06, 116.61) | <0.0001 | 1.08(1.05, 1.11) | <0.0001 |
| Heart failure | 64.98(64.72, 65.24) | 74.59(74.16, 75.02) | 78.07(77.41, 78.73) | 95.38(94.17, 96.6) | <0.0001 | 1.20(1.16, 1.24) | <0.0001 |
| Ischemic heart disease | 229.85(229.38, 230.31) | 283.54(282.76, 284.32) | 323.35(322.16, 324.54) | 368.88(367.02, 370.76) | <0.0001 | 1.41(1.38, 1.44) | <0.0001 |

Values are age-adjusted mortality rates per 100,000 adults, with 95% confidence intervals (CIs) provided in parentheses.

* P value for trend was derived from generalized linear models (GLMs) by treating the quartile categories as a continuous ordinal variable, testing for a linear gradient in CKM mortality rates across increasing levels of exercise access (from Q1 to Q4).

† P value presented alongside the rate ratio was obtained from the same GLMs, specifically testing the null hypothesis that the rate ratio equals 1.00 (i.e., no difference in mortality risk between the highest and lowest quartiles of exercise access).

‡ All models were adjusted for the following covariates: year, percentage of female, percentage of Hispanic, percentage of rural population, percentage of population over the age of 65 years, percentage of uninsured population, ratio of population to primary care physician, household income inequality, adults smoking prevalence, preventable hospital stay rate, and food environment index. This adjustment set corresponds to the fully adjusted specification (Model 2) employed in the subsequent regression analyses.
