# Supplementary material for: Associations of access to exercise opportunities and physical inactivity with cardiovascular–kidney–metabolic mortality: a nationwide ecological study
Source: Front Public Health. 2026 Oct 1;14:1924014. doi: 10.3389/fpubh.2026.1924014 (PMC13668831; doi:10.3389/fpubh.2026.1924014)
Supplement: Supplementary file 8 [file Table_2.DOCX]

**Supplementary Table S2. Percentage of Counties in the Extreme Quartiles of Each Indicator by U.S. Census Region, 2014 and 2019**

| **Region** | **Year** | **ATEO Q1 (lowest access)** **, %** | **PI Q4 (highest inactivity)** **, %** | **AAMR Q4 (highest mortality)** **, %** |
| --- | --- | --- | --- | --- |
| **South** | 2014 | **45.1** | **43.6** | **39.9** |
|  | 2019 | **25.1** | **28.6** | **35.6** |
| **Midwest** | 2014 | 26.6 | 18.3 | 16.3 |
|  | 2019 | 12.6 | 6.7 | 13.9 |
| **West** | 2014 | 25.8 | 2.5 | 4.5 |
|  | 2019 | 9.6 | 0.0* | 5.0 |
| **Northeast** | 2014 | 8.0 | 4.1 | 2.8 |
|  | 2019 | 2.8 | 1.3 | 2.3 |

*West had zero counties in the highest PI quartile (Q4) in 2019.
