# Supplementary material for: Associations of access to exercise opportunities and physical inactivity with cardiovascular–kidney–metabolic mortality: a nationwide ecological study
Source: Front Public Health. 2026 Oct 1;14:1924014. doi: 10.3389/fpubh.2026.1924014 (PMC13668831; doi:10.3389/fpubh.2026.1924014)
Supplement: Supplementary file 9 [file Table_3.DOCX]

**Table S3.** Model diagnostics for negative binomial regression models with clustered standard errors and spatial sensitivity analysis.

| **Exposure** | **Dispersion (Theta)** | **RR (Q4 vs Q1, 95% CI)** | **Absolute Rate Diff. (ARD) per 100k** | **Moran's I on Residuals** | **SEM Sensitivity (Coefficient for Exposure)** |
| --- | --- | --- | --- | --- | --- |
| **Physical Inactivity** | 42.07 | 1.23 (1.22 – 1.25) | +112.38 | 0.219 (P < 0.001) | 0.116 (P < 0.001) |
| **Access to Exercise**  **Opportunities** | 35.93 | 0.89 (0.87 – 0.90) | –78.81 | 0.219 (P < 0.001) | –0.018 (P = 0.077) |
